# Orbital Lymphangioma in an Adolescent Male: A Case Report and Review of Literature

**DOI:** 10.7759/cureus.74813

**Published:** 2024-11-30

**Authors:** Satyanarayana Kummari, Mahipal Ranga

**Affiliations:** 1 Radiodiagnosis, All India Institute of Medical Sciences, Nagpur, Nagpur, IND; 2 Radiodiagnosis, MNR Medical College and Hospital, Sangareddy, IND

**Keywords:** chemosis, intralesional bleomycin sclerotherapy, keratopathy, lymphangiomas, monocular diplopia, mri-magnetic resonance imaging, ocular motility disorders. amblyopia, unilateral proptosis

## Abstract

Lymphangiomas are localized multi-cystic malformations of the lymphatic and vascular system, primarily affecting the head and neck regions in children. Orbital lymphangiomas are not considered hamartomas because the orbit does not commonly display lymphatic vessels. In this case report, we describe a male patient who was 15 years old and presented to our medical facility with the primary complaints of having a bulging left eye, sudden chemosis of the lower conjunctiva, and pain in the left eye. During the examination of the left eye by the ophthalmologist, proptosis, hyperlacrimation, conjunctival hyperemia, keratopathy, and pain on probing were found. A magnetic resonance imaging (MRI) of both orbits was recommended, which revealed an ill-defined, multi-locular heterogeneous solid-cystic mass in the left retrobulbar space. He was subsequently admitted to the healthcare facility for a biopsy of the orbital lesion. The examination of the specimen, both at the macroscopic and microscopic levels, was consistent with the diagnosis of lymphangioma. Given the potential risk of damage to adjacent structures, the patient was initially scheduled for sclerotherapy rather than a surgical excision. A total of three sessions of bleomycin-based sclerotherapy were administered to the patient. The patient gradually improved in the follow-up period, achieving complete remission of pain, proptosis, and chemosis. However, considering the possibility of new episodes arising, it was recommended that he attend regular follow-up appointments. The purpose of this case report is to present a rare case of orbital lymphangioma and emphasize the clinical and radiological features and management of orbital lymphangiomas.

## Introduction

Lymphangiomas are localized multi-cystic malformations of the lymphatic and vascular system, primarily affecting the head and neck regions in children. Orbital lymphangiomas are very rare, accounting for approximately 0.3%-4% of all orbital tumors. Orbital lymphangiomas are not considered hamartomas because the orbit does not commonly display lymphatic vessels [1].

Clinically, it can present as proptosis, restricted movement of the eye, ptosis, ocular pain, diplopia, corneal exposure, and visual impairment resulting from amblyopia and compression of the optic nerve. There is a tendency for the clinical manifestations to become more severe in instances of internal hemorrhage into the lesion and bouts of upper airway infection. These medical conditions lead to lymphoid hyperplasia as a consequence of a response of the immune system, which could account for the re-emergence of the condition [2].

Early diagnosis and treatment are absolutely necessary to preserve vision and prevent amblyopia in patients with orbital lymphangiomas. Radiological imaging is the most effective method for confirming the diagnosis of lymphangiomas, and it allows for a simultaneous evaluation of its dimensions and extension [3,4]. Lymphangiomas frequently involve essential orbital structures; hence, surgical procedures such as debulking or total excision may not be feasible since they have the potential to cause damage to vital structures that are located nearby. In addition, there is a considerable risk of scarring and recurrence associated with surgical procedures. Sclerotherapy serves as an alternate method of management that has shown promising results in these cases [5].

## Case presentation

**Figure 1:**
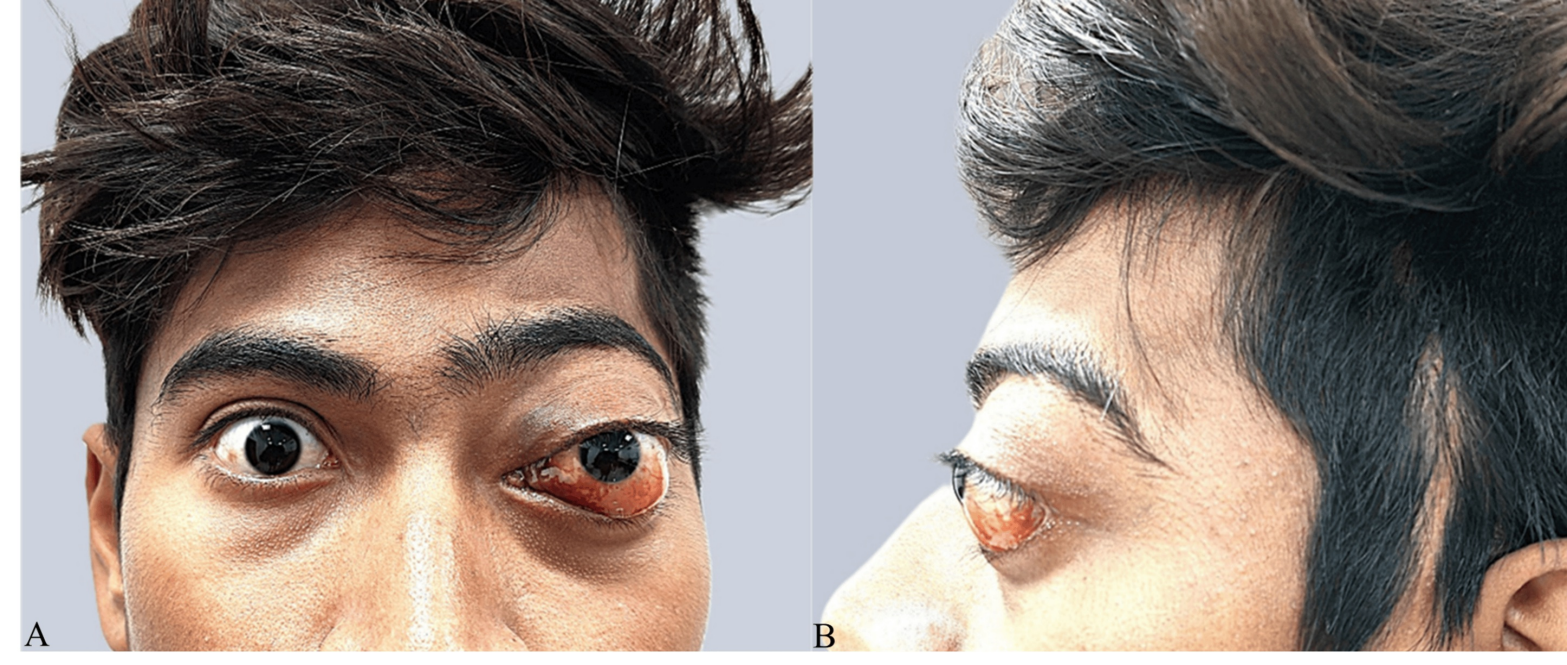
Clinical photographs (A, B) show proptosis of the left eye associated with conjunctival hyperemia, and keratopathy

A male patient who was 15 years old presented to our medical facility with the primary complaints of having a bulging left eye, sudden chemosis of the lower conjunctiva, and pain in the left eye. In addition, the patient suffered a dry cough, a running nose, a decrease in appetite, and a loss of weight. The patient was irritable, feeble, and drained when the clinical examination was performed. During the examination of the left eye by the ophthalmologist, proptosis, hyperlacrimation, conjunctival hyperemia, keratopathy, and pain on probing were found (Figure 1). An increase in tortuosity of the blood vessels and distortion of the optic disc were observed during the fundoscopy examination.

**Figure 2:**
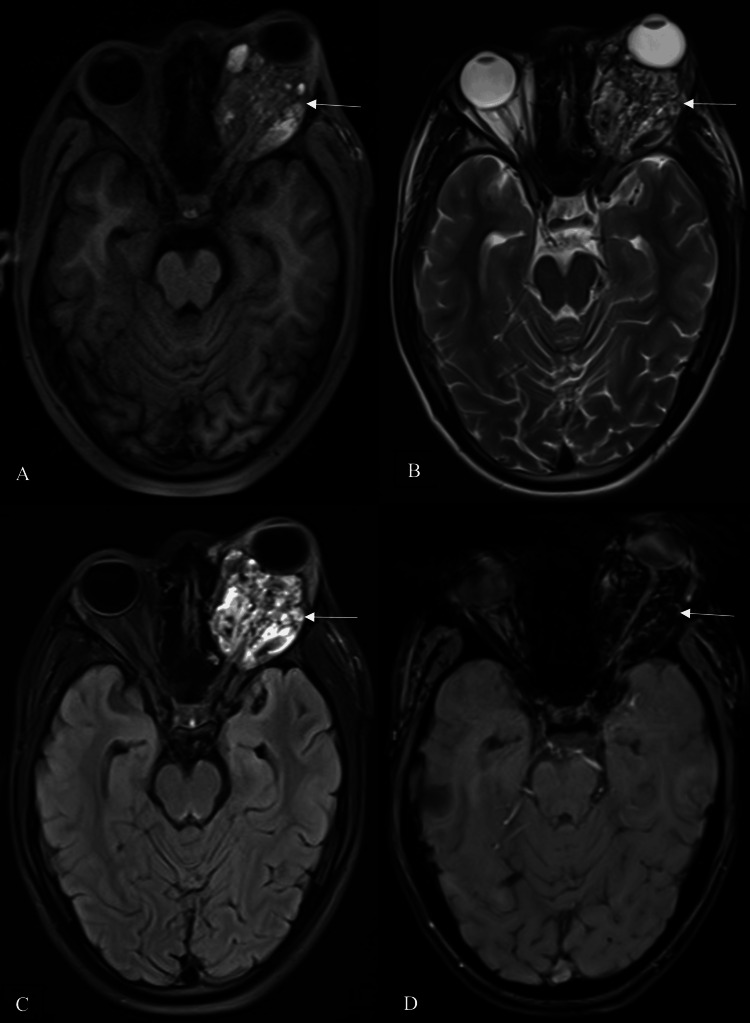
MRI of both orbits. A: T1WI, B: T2WI, C: FLAIR, D: GRE Axial images show an ill-defined, multi-locular heterogeneous solid-cystic mass in the left retrobulbar space. The lesion exhibits an isointense to hyperintense signal on T1WI, a hyperintense signal on T2WI and FLAIR sequences with fluid-fluid levels, and blooming on the GRE sequence MRI: Magnetic resonance imaging, FLAIR: Fluid attenuated inversion recovery, GRE: Gradient echo

A magnetic resonance imaging (MRI) of both orbits was recommended, which revealed an ill-defined, multi-locular heterogeneous solid-cystic mass in the left retrobulbar space. This mass involves the intra- and extraconal spaces surrounding the optic nerve and extends to the upper and lower eyelids and also to the frontal region, accompanied by bone remodeling. The lesion exhibits an isointense to hyperintense signal on T1-weighted imaging and a hyperintense signal on T2-weighted imaging, with numerous T1 and T2 hyperintense areas and fluid-fluid levels prominently observed on T2-weighted imaging. The post-contrast T1 weighted imaging reveals a heterogeneous enhancement in the lesion. The lesion exerts a mass effect on the orbital structures, primarily affecting the optic nerve and the eye globe, leading to visual disturbances and proptosis (Figure 2). 

He was subsequently admitted to the healthcare facility for a biopsy of the orbital lesion. The examination of the specimen, both at the macroscopic and microscopic levels, was consistent with the diagnosis of lymphangioma. Given the potential risk of damage to adjacent structures, the patient was initially scheduled for sclerotherapy rather than a surgical excision. A total of three sessions of bleomycin-based sclerotherapy were administered to the patient. Following the third sclerotherapy treatment, the pain, proptosis, and chemosis had considerably decreased. 

To assess the impact of sclerotherapy sessions, a follow-up orbital MRI was done, and the results indicated a substantial reduction in the size of the lesion. The patient gradually improved in the follow-up period, achieving complete remission of pain, proptosis, and chemosis. The most recent assessment took place after a two-month interval, showing no complaints and no signs of proptosis. However, considering the possibility of new episodes arising, it was recommended that he attend regular follow-up appointments. 

## Discussion

Lymphangiomas, commonly referred to as lymphatic malformations, are localized, multicystic abnormalities that affect the vascular and lymphatic systems. It has been determined that there are between 1.1 and 5.3 cases of lymphangioma for every 10,000 live births. Approximately 25% of the benign vascular tumors in children are caused by lymphangiomas, which account for 4% of all vascular tumors. Lymphangioma of the orbit is regarded as extremely uncommon, accounting for approximately 1-4% of all orbital lesions [6]. Hereditary or acquired abnormalities of the lymphatic system can lead to the development of lymphangiomas. The congenital type, which typically manifests before a youngster turns five, is caused by improper communication of the lymphatic ducts with the primary lymphatic duct. The development of acquired lymphangioma can be attributed to a number of factors, including malignancies, radiation therapies, trauma, or surgeries [7,8]. 

The most prevalent symptoms of orbital lymphangiomas are ptosis and proptosis, and most of them occur during the first ten years of life [1]. Typically, such lesions remain unrecognized in clinical settings until intralesional hemorrhage occurs, leading to acute proptosis. This occurrence is typical in infants as they grow more active and susceptible to trivial injuries. In cases where an infection is present in the upper respiratory tract, the lesions typically exhibit a markedly enlarged appearance. At times, the lesion may lead to a gradual increase in proptosis and limitations in ocular movement. It is possible that localized lesions will not present any symptoms and will only be identified by chance during neuroradiological imaging in extremely rare instances. Primarily following acute hemorrhage into the lesion, lesions can cause mass effects on adjacent structures and induce compressive optic neuropathy. The identification of such possible amblyogenic factors is crucial, as patients often arrive prior to complete visual maturation, which may necessitate intervention with surgery [1,9]. 

In most cases, orbital lymphatic malformations involve all of the orbital spaces, including the preseptal, postseptal, intraconal, and extraconal spaces. They are ill-defined, non-encapsulated, heterogenous multilocular solid-cystic lesions. CT imaging can reveal the presence of phleboliths in the lesion along with accompanying bony remodeling that occurs as a result of the compressive impact of the lesion on nearby bony structures. The MRI scan outperforms a CT scan in evaluating such lesions, providing an excellent assessment of their extent and illustrating numerous components effectively. The MRI scan has been proven to detect feeder blood vessels that are difficult to detect on CT scans. It also exposes acute and chronic hemorrhages within the lesion. The existence of these feeding blood vessels sets this lesion apart from other vascular lesions in the orbit. It has been demonstrated through angiographic investigations that the lesion is separate from both the arterial and venous circulations. It was found that lesions branch into the structures that were surrounding them without invasion of these structures [1,10].

Lymphangiomas present significant management challenges due to their potential to induce structural as well as functional disorders, which are influenced by factors such as location, size, and their interactions with surrounding structures. In spite of the fact that conservative observation is frequently recommended for asymptomatic patients, early intervention may be considered due to the fact that lymphatic venous malformations have a tendency to increase with advancing age. Lesions that could cause permanent loss of vision must be treated immediately. Both surgical and nonsurgical approaches were considered for treatment [6]. Non-surgical management alternatives encompass observation, sclerotherapy, and medication-based therapies. Observation of the lesion may be appropriate for patients who do not exhibit any signs and symptoms that threaten the vision or have significant functional limitations. Medications like PDE 5 inhibitors (Sildenafil) or mTOR inhibitors (Bleomycin and Sirolimus) can be utilized in medical therapy. Alternative approaches for managing these lymphangiomas encompass debulking and/or surgical excision; nonetheless, due to the lesions' frequent proximity to or entanglement with essential structures in the orbit, surgical intervention is often challenging. Moreover, the likelihood of recurrence remains significant. In light of the associated risks, surgical excision remains the primary approach for managing orbital lymphangiomas [4,8,11].

## Conclusions

Lymphangiomas are localized multi-cystic malformations of the lymphatic and vascular system, primarily affecting the head and neck regions in children. Orbital lymphangiomas are extremely uncommon orbital tumors. Early diagnosis and treatment are absolutely necessary to preserve vision and prevent amblyopia in patients with orbital lymphangiomas. Radiological imaging is the most effective method for confirming the diagnosis of lymphangiomas. Surgical interventions such as debulking and/or surgical excision can frequently pose challenges due to the frequent proximity of lesions to vital orbital structures or their entanglement with them. Furthermore, there is a substantial probability of a recurrence. Sclerotherapy is an effective, minimally invasive alternative treatment for orbital lymphangiomas that offers favorable outcomes.
